# Effect of Sodium Selenite on Pathological Changes and Renal Functions in Broilers Fed a Diet Containing Aflatoxin B_1_

**DOI:** 10.3390/ijerph120911196

**Published:** 2015-09-09

**Authors:** Na Liang, Fengyuan Wang, Xi Peng, Jing Fang, Hengmin Cui, Zhengli Chen, Weimin Lai, Yi Zhou, Yi Geng

**Affiliations:** Key Laboratory of Animal Diseases and Environmental Hazards of Sichuan Province, College of Veterinary Medicine, Sichuan Agricultural University, Wenjiang 611130, China; E-Mails: liangna19911314@163.com (N.L.); wfy_sccd@163.com (F.W.); cui580420@sicau.edu.cn (H.C.); chzhli75@163.com (Z.C.); nwm_mm2004@163.com (W.L.); 13981616210@139.com (Y.Z.); gengyisicau@126.com (Y.G.)

**Keywords:** aflatoxin B_1_, sodium selenite, pathological observation, ultrastructural observation, renal function, broiler

## Abstract

To evaluate the renal toxicity of dietary aflatoxin B_1_ (AFB_1_) and ameliorating effects of added dietary sodium selenite in broiler, renal histopathological changes, ultrastructural changes, and renal function parameters were monitored at 7, 14, and 21 days of age. Two hundred one-day-old healthy male Avian broilers were divided into four groups, namely control group, AFB_1_ group (0.3 mg/kg AFB_1_), +Se group (0.4 mg/kg Se), and AFB_1_+Se group (0.3 mg/kg AFB_1_+0.4 mg/kg Se). Compared with that of the control group, the relative weight of kidney was increased in the AFB_1_ group. There were no significant differences between the AFB_1_+Se group and the control group. By histopathological observation, the renal epithelia were swelling and necrosis at 7 and 21 days of age. Ultrastructurally, the lipid droplets and expanded endoplasmic reticulum appeared in the plasma of epithelia cells in the AFB_1_ group. Enlarged mitochondria with degenerated cristae were observed in the +Se group. Compared with the control group, the contents of serum creatinine and serum uric acid in the AFB_1_ group were increased, while the activity of renal Na^+^-K^+^ ATPase was decreased. When 0.4 mg/kg selenium was added into the diet containing 0.3 mg/kg AFB_1_, there were no obvious histological changes in the AFB_1_+Se group, and the contents of the serum creatinine and serum uric acid contents and the activity of renal Na^+^-K^+^ ATPase were close to those in the control group. In conclusion, sodium selenite exhibited protective effects on AFB_1_-induced kidney toxicity in broilers.

## 1. Introduction

Aflatoxins (AFs) are potent carcinogens that are produced as secondary metabolites of strains of the fungi *Aspergillus parasiticus* , *Aspergillus nomius*, and *Aspergillus flavus* that contaminate agricultural commodities before or under post-harvest conditions [1]. Among a number of confirmed aflatoxins, aflatoxin B_1_ (AFB_1_) is the normally predominant and most toxic form [2]. It was well documented that AFB_1_-contaminated products may result in inhibition of growth performance, lesions of liver and kidney, and suppression of immune function, and so on [3,4,5]. Though AFB_1_ is firstly found to be a hepatotoxin, it also could cause damages to renal tissue and reduce renal function *in vitro* and *in vivo* [6].

Although a high level of selenium (Se) was toxic to animals, appropriate Se functions as an essential micronutrient in the animal diet. Se is incorporated as selenocysteines, some of which perform important enzymatic functions [7]. Se-deficiency diseases have been identified as reproductive impairment and growth depression [8], and White Muscle Disease [9] in animals. Selenium-containing compounds are important for the health of human beings and animals [10,11]. Appropriate Se could also protect against hepatocellular oxidative damages caused by lipopolysaccharide [12], jejunal apoptosis resulted from AFB_1_ [13], and renal damages induced by drugs [14] and some metallic element [15].

The kidney serves several essential roles, including producing urine, filtering blood, and removing harmful waste products [16]. As previous study reported, AFB_1_ decreased glomerular filtration rate, tubular reabsorption of glucose, and tubular transport for p-aminohippurate [17], and even cause tumors in kidney [18]. AFB_1_ treatment increases the relative weight of the kidney [6] which may be partly related to the presence of vacuolar degeneration of the renal tubules [19]. AFB_1_ exposure may markedly increase the level of lipid peroxide, decrease the activities of antioxidase [20], and decrease sodium-phosphate uptake [21]. However, dietary addition of selenium yeast partially counteracted the negative effects of AFB_1_ on weight gain and death rate [22]. Dietary Se could relieve liver lesion [23] and immunosuppression [24] induced by AFB_1_, and could protect renal cells from oxidative stress *in vitro* [25], and inhibit AFB_1_-induced apoptosis and cell cycle blockage in renal cells of broiler [26].

Our earlier studies demonstrated that 0.3mg/kg AFB_1_ in diet had obvious adverse effects on broilers, and appropriate level of Se supplied in the diet (0.4 mg/kg) could provide optimal protective effects against AFB_1_-induced toxicity in broilers [27,28]. Based on this information, toxin concentrations (0.3mg/kg AFB_1_) and supplemented Se levels (0.4 mg/kg) were chosen in our present research, and sodium selenite was chosen as the source of supplemented Se. In order to evaluate the effects of dietary sodium selenite on AFB_1_-induced lesions of kidney, relative weight, pathological and ultrastructural changes of kidney, the contents of serum creatinine and uric acid and the activity of renal Na^+^-K^+^ ATPase were determined. The relationship of these parameters could deepen the knowledge on the mechanisms of renal lesions induced by AFB1 exposure.

## 2. Materials and Methods

### 2.1. Chickens and Diets

Two hundred one-day-old healthy male Avian broilers were purchased from a commercial rearing farm (Wenjiang poultry farm, Sichuan province, China) and randomly divided into four equal groups of three pen replicates, namely control group, AFB_1_ group (0.3 mg/kg AFB_1_), +Se group (0.4 mg/kg Se) and AFB1+Se group (0.3 mg/kg AFB1+0.4 mg/kg Se). Birds of each experimental group were housed in three cages with electrically-heated units and were provided with water, as well as aforementioned diets, *ad libitum* for 21 days. AFB_1_ farinose solid (3 mg) was completely dissolved in methanol (30 mL), and then the 30 mL mixture was mixed into 10 kg corn-soybean basal diet to formulate the AFB_1_ diet of experimental groups containing 0.3 mg/kg AFB_1_. The equivalent methanol was mixed into the corn-soybean basal diet to produce the control diet, then the methanol of the diets was evaporated at 98 °F (37 °C) [13]. By hydride-generation atomic absorption spectroscopy, the content of dietary Se in the control group was 0.332 mg/kg. Thus, the concentration of Se in each group was: 0.332 mg/kg (control group), 0.332 mg/kg (AFB_1_ group), 0.732 mg/kg (+Se group), and 0.732 mg/kg (AFB_1_+Se group), respectively. Aflatoxin B_1_ (AFB_1_) was obtained from Pribolab Pte. Ltd (Singapore, Singapore). All experimental procedures involving animals were approved by Sichuan Agricultural University Animal Care and Use Committee. Nutritional requirements were adequate according to National Research Council (1994) [29] and Chinese Feeding Standard of Chicken (NY/T33-2004).

### 2.2. Relative Weight of Kidney

At 7, 14, and 21 days of age during the experiment, after the body weight was weighed, six birds in each group were euthanized and necropsied. Kidney was dissected from each chick and weighed after dissecting connective tissue around the organ. Relative weight of kidney was calculated by the following formula:

Relative weight = organ weight (g)/body weight (kg)
(1)

### 2.3. Histopathological Observation

After weighing, kidneys were fixed in 4% paraformaldehyde for more than 24 h and routinely processed in graded alcohol, and then embedded in paraffin. Thin sections (5 μm) of each tissue were sliced and mounted on glass slide. Slides were stained with hematoxylin and eosin Y. Histological slides were examined on an Olympus light microscope. Histologic lesions were took photographs by Nicon micrographic system (Nicon, Tokyo, Japan).

### 2.4. Ultrastructural Observation

At 21 days of age during the experiment, three broilers in each group were euthanized and then immediately necropsied. The kidney of each broiler was dissected and then fixed in 2.5% glutaraldehyde for 48 h. After rinsed with phosphate buffer solution, the tissues were postfixed in 2% Veronal acetate-buffered O_s_O_4_ for 2 h. After dehydrated in graded alcohol, they were embedded in Araldite. Processing of renal slides for ultrastructural observation was performed according to Reynolds [30]. After obtained by Reichert-Jung Ultracut E (Leica, Germany, UC7), thin sections (70 nm) were stained with uranyl acetate and 0.2% lead citrate. After that, the samples were examined with a electron microscope (Hitachi H-600, Hitachi, Japan).

### 2.5. Detection of Serum Creatinine and Uric Acid

At 7, 14, and 21 days of the experiment, six chickens in each group were chosen and blood samples were collected through the jugular vein. The serum of each bird was analyzed for detecting the contents of serum creatinine and uric acid according to kit instructions (Jiancheng, Nanjing, China).

### 2.6. Detection of Na^+^-K^+^ ATPase in Kidney

At 7, 14, and 21 days of the experiment, six chickens in each group were euthanized and the renal tissues were immediately collected for detecting the activity of Na^+^-K^+^ ATPase. 1 g renal tissue was homogenized with 9 mL normal saline buffer through cell homogenizer in ice bath and centrifuged at 3000 r/min for 10 min to obtain a clear supernatant. The type of centrifuge was TD24-WS of Xiangyi Co. in China. After determining the amount of total protein in the supernatant of the renal homogenate by the method of Bradford (1976) [31], the Na^+^-K^+^ ATPase activities was measured by biochemical method following the instruction of reagent kits (Jiancheng, Nanjing, China).

### 2.7. Statistical Analysis

Statistical analysis was performed with SPSS software for Mac v16.0 (IBM Corp, Armonk, NY, USA). All parameters determined in this study were presented as mean ± standard deviation (mean ± SD). Statistical analyses were performed using one-way analysis of variance or t-test, and Dunnett’s T3 test was employed for multiple comparisons, differences were considered significant at *p* < 0.05 and very significant at *p* < 0.01.

## 3. Results

### 3.1. Changes of Relative Weight of Kidney

No significant differences were observed among the four groups at 7 and 14 days of age. At 21 days of age, the relative weight of kidney in the AFB_1_ group was increased (*p* < 0.05) when compared with those in the control group and +Se group. However, there were no significant differences between the AFB_1_+Se group and the control group during the experiment. The results were shown in Table 1.

**Table 1:** Relative weight of kidney (g/kg).

Group	7 Days of Age	14 Days of Age	21 Days of Age
Control group	10.121 ^a^ ± 0.284	8.581 ^a^ ± 1.054	6.754 ^c^ ± 0.608
AFB_1_ group	10.250 ^a^ ± 0.257	8.413 ^a^ ± 0.749	7.355 ^a^ ± 0.460
+Se group	10.087 ^a^ ± 0.413	8.895 ^a^ ± 0.404	6.819 ^bc^ ± 0.346
AFB_1_+Se group	10.057 ^a^ ± 0.912	8.674 ^a^ ± 0.572	6.949 ^abc^ ± 0.226

Note: Data are presented with the means ± standard deviation (*n* = 5). The difference between data with different lowercase letters (a–c) within a column is significant (^a,b,c^ *p* < 0.05).

### 3.2. Pathological Lesions

**Figure 1:**
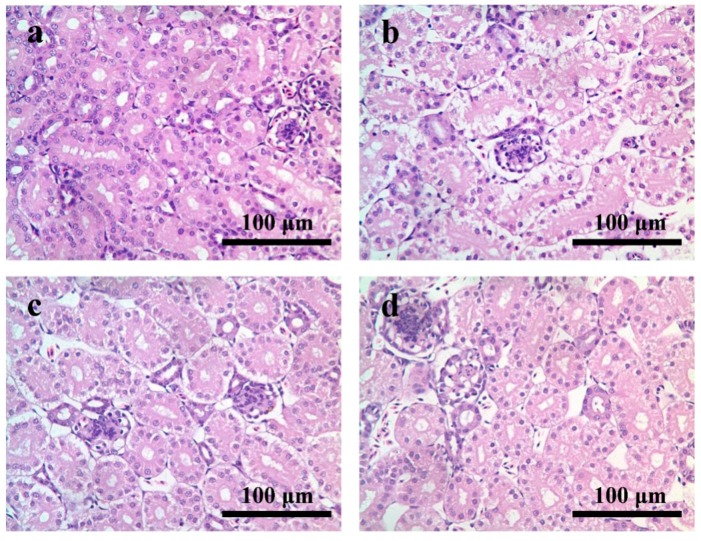
(**a**) Kidney of the 7-day-old in control group; (**b**) kidney of the 7-day-old in AFB_1_ group: the renal cells were swelling and the renal tubules cavity seriously closure; (**c**) kidney of the 7-day-old in +Se group; (**d**) kidney of 7-day-old in AFB_1_+Se group. Bars = 100 μm.

In the control group, the histological structure was normal, which showed as normal renal tubular epithelial cells with homogeneous plasma, and obvious renal tubular cavity (Figure 1a). At 7 days of age, compared with the control group, the renal cells were degenerated, and the renal tubule’s cavity was almost closed in the AFB_1_ group. The renal tubular epithelium were mainly granular degeneration or vesicular degeneration, which sometimes showed as hydropic degeneration and sometimes appeared as fatty degeneration with suborbicular small vacuoles in the cytoplasm (Figure 1b). In the +Se group, the renal tubular epithelium was mainly granular degeneration (Figure 1c). There were slightly granular degeneration of the renal tubular epithelium in the AFB_1_+Se group (Figure 1d).

**Figure 2:**
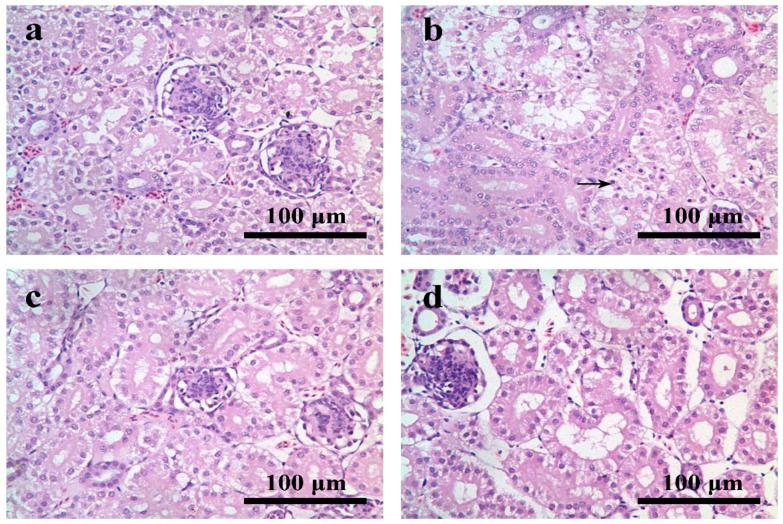
(**a**) Kidney of the 21-day-old in control group; (**b**) kidney of the 21-day-old in AFB_1_ group: a great number of necrotic cells was observed in renal tubular epithelial cells (→); (**c**) kidney of the 21-day-old in +Se group; (**d**) kidney of 21-day-old in AFB_1_+Se group. Bars = 100 μm.

At 14 and 21 days of age, compared with the control group (Figure 2a), the renal tubular epithelial cells in partial regions were necrotic in the AFB_1_ group. The necrotic cells appeared pyknotic with shrinking and dark dyeing nuclei and a lighter color of the plasma when compared with the surrounding normal renal tubule (Figure 2b). In the +Se and AFB_1_+Se groups, the kidneys had no obvious changes when compared with the control group (Figure 2c,d).

### 3.3. Electron Microscopic Appearance

**Figure 3:**
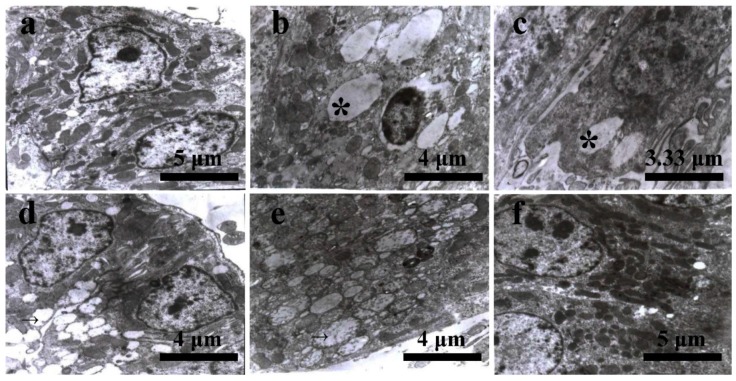
(**a**) Kidney of the 21-day-old in control group: proximal convoluted tubular epithelial cell with a great numbers of mitochondria and rough endoplasmic reticulum; (**b**) kidney of the 21-day-old in AFB_1_ group: lipid droplets appeared (*); (**c**) kidney of 21-day-old in AFB_1_ group: lipid droplets appeared (*); (**d**) kidney of the 21-day-old in +Se group: The mitochondria were swollen and enlarged (→); (**e**) kidney of the 21-day-old in +Se group: The cristae of mitochondria were irregularly ranked and degenerated (→); (**f**) kidney of the 21-day-old in AFB_1_+Se group: renal epithelial cell with a great number of normal mitochondria.

At 21 days of age, the ultrastructure of kidneys in the control group appeared normal. In the cytoplasm of proximal convoluted tubular epithelial cells, a great number of mitochondria and rough endoplasmic reticulum were observed (Figure 3a). In the AFB_1_ group, affected cells were mainly renal tubular epithelial cells, in which the endoplasmic reticulum expanded and a lot of lipid droplets appeared (Figure 3b). The lipid droplets were also observed in the cytoplasm of vascular epithelial cells (Figure 3c). In the +Se group, the mitochondria in renal tubular epithelial cells were swollen and enlarged (Figure 3d) with irregularly ranked and degenerated cristae (Figure 3e). In the AFB_1_+Se group, the ultrastructure of kidneys appeared normal. The normal renal tubular epithelial cells with a lot of mitochondria were observed (Figure 3f).

### 3.4. Changes of Serum Creatinine and Uric Acidcontents

By biochemical detection, from 7 to 21 days of age, the contents of serum creatinine were higher (*p* < 0.05 or *p* < 0.01) in the AFB_1_ group than those in the control group, and there were no significant differences between the +Se group and the control group. However, when compared with those in the AFB_1_ group, the content of serum creatinine in the AFB_1_+Se group were decreased (*p* < 0.05) at 7 and 14 days of age, and was evidently decreased (*p* < 0.01) at 21 days of age.

From 7 to 21 days of age, the contents of serum uric acid in the AFB_1_ group were increased (*p* < 0.05) when compared with those in the control group. Meanwhile, there were no significant differences between +Se group and control group from 7 to 21 days of age. Compared with those in the AFB_1_ group, the contents of serum uric acid in AFB_1_+Se group were decreased (*p* < 0.05) from 7 to 21 days of age. The results were shown in Table 2.

**Table 2:** Changes of serum creatinine and uric acid.

Content	Group	7 Days of Age	14 Days of Age	21 Days of Age
Creatinine (μmol/L)	Control group	1206.126 ^d^ ± 114.155	1029.423 ^d^ ± 86.673	1173.729 ^bc^ ± 82.053
	AFB1 group	1373.289 ^a^ ± 50.225	1277.720 ^a^ ± 106.370	1298.965 ^a^ ± 104.439
	+Se group	1210.832 ^bcd^ ± 121.373	1103.771 ^bcd^ ± 160.967	1199.336 ^bc^ ± 23.058
	AFB1+Se group	1208.176 ^cd^ ± 122.126	1063.861 ^cd^ ± 96.860	1142.524 ^c^ ± 80.103
Uric acid (mg/L)	Control group	55.540 ^bc^ ± 7.085	55.706 ^c^ ± 5.773	58.626 ^d^ ± 6.095
	AFB1 group	66.761 ^a^ ± 10.601	67.441 ^a^ ± 6.320	69.272 ^a^ ± 6.682
	+Se group	59.932 ^abc^ ± 5.861	58.804 ^abc^ ± 6.687	59.657 ^bcd^ ± 6.303
	AFB1+Se group	54.000 ^c^ ± 7.175	57.538 ^bc^ ± 9.707	59.089 ^cd^ ± 7.509

Note: Data are presented with the means ± standard deviation (*n* = 5). The difference between data with different lowercase letters (a–d) within a column is significant (^a,b,c,d^ *p* < 0.05).

### 3.5. Changes of Na^+^-K^+^ ATPase in Kidney

At 7 and 21 days of age, the activities of Na^+^-K^+^ ATPase in the AFB_1_ group were lower (*p* < 0.05 or *p* < 0.01) than those in the control group. There were no significant differences between the +Se group and the control group from 7 to 21 days of age. Compared with those in the AFB_1_ group, the activities of Na^+^-K^+^ ATPase in the AFB_1_+Se group were increased (*p* < 0.05) from 7 to 21 days of age. The results were shown in Table 3.

**Table 3:** Changes of the activity of Na^+^-K^+^ ATPase in kidney.

Group	7 Days of Age	14 Days of Age	21 Days of Age
Control group	9.661 ^a^ ± 0.830	9.378 ^ab^ ± 0.761	11.947 ^a^ ± 2.030
AFB1 group	8.125 ^b^ ± 1.208	8.264 ^b^ ± 1.322	9.007 ^b^ ± 1.127
+Se group	9.118 ^ab^ ± 1.109	9.280 ^ab^ ± 0.888	11.407 ^a^ ± 2.137
AFB1+Se group	9.675 ^a^ ± 0.642	9.552 ^a^ ± 1.065	11.526 ^a^ ± 1.624

Note: Data are presented with the means ± standard deviation (*n* = 5). The difference between data with different lowercase letters (a,b) within a column is significant (^a,b^ *p* < 0.05).

## 4. Discussion

As we known, the kidney serves several essential roles including producing urine, filtering blood, and removing harmful waste products to maintain organism homeostasis [32]. In our present study, the intake of AFB_1_ could increase the relative weight of kidney in chickens. This result is in accordance with previous researches, which showed that an AFB-contaminated diet could increase the relative weight of kidney in broilers [6,33].

The histopathological lesions of kidney in the AFB_1_ group include swelling and necrosis of renal epithelium. The cell swelling was appeared at 7 days of age, and the necrosis of renal tubular epithelia was observed at 21 days of age. The gradually deteriorated lesions may be caused by the accumulation of AFB_1_ metabolites. The cell swelling (including granular degeneration and hydropic degeneration) of tubular epithelium might be related to the increased relative weight in the AFB_1_ group. Ultrastructurally, lipid droplets apprearance and endoplasmic reticulum enlargement in renal tubular epithelial cells were observed in the AFB_1_ group. Those lesions were coincidence with the vesicular degeneration of renal tubular epithelial cells in histological observation.

The activities of Na^+^-K^+^ ATPase in the AFB_1_ group were lower than those in the control group, and markedly increased contents of serum creatinine and serum uric acid were observed. The decrease of Na^+^-K^+^ ATPase activity may be caused by the combination of AFB_1_ with Na^+^- and K^+^-activated sites [34], which affects exchange of Na^+^ and K^+^ in sodium pomp, and the ion exchange imbalances would accordingly cause sodium and water retention in cells [35]. Thus, the increase of lipid droplets in cytoplasm and the enlargement of endoplasmic reticulum might be concerned with the cell metabolism disorders induced by the decline of Na^+^-K^+^ ATPase activity. The obvious cell swelling could induce the occlusion of the renal tubule and then could gradually decrease the excretory function of the kidney. Therefore, the increased contents of serum creatinine and serum uric acid were deserved in the AFB_1_ group, which was in accordance with Mathuria’s study that an elevation of creatinine in serum was observed in mice receiving aflatoxin-contaminated feeds [36] and Santurio’s observation that serum uric acid content increased in broilers intaking AFB_1_ [37]. The histopathological results showed an occlusion of the renal tubule, which could be a causal relationship in the increase of serum creatinine and uric acid contents. The increased contents of serum creatinine and uric acid may be resulted from the excretion disorder of kidney because the kidney rapidly excretes creatinine and uric acid in avians [38].

The kidney is critical for the urinary system and serves homeostatic functions [32]. Previous studies found AFB_1_ could increase relative weight of kidney, decrease renal glomerular filtration rate, tubular reabsorption of glucose tubular transport for p-aminohippurate [17], urine flow rate, sodium/potassium excretion, and increasing urine hydrogen ion concentration [39]. In our present study, the inhibition of relative weight of kidneys, an increased content of serum creatinine and uric acid, and the decline of activities of Na^+^-K^+^ ATPase were observed in the AFB_1_ group. These results indicated that 0.3 mg/kg AFB_1_ could damage the renal function in broilers.

However, Se could effectively protect kidneys from adverse effects caused by AFB_1_. Previous studies showed that 0.267 mg/kg–0.6 mg/kg Selenium supplemented could ameliorate the damages induced by AFB_1_ to some extent [28,40,41]. In our present study, when 0.4 mg/kg selenium was added into the AFB_1_ diet, the AFB_1_-associated lesions of kidneys, including the increased renal relative weight, pathological and ultrastructural changes, the increased serum contents of creatinine and uric acid, and the decreased activities of renal Na^+^-K^+^ ATPase were all ameliorated, and there were no significant differences for these parameters between the AFB_1_+Se group and the control group. The possible mechanisms of protective role of sodium selenite might be associated with following factors: (1) Se was the nucleus of antioxidant selenoenzymes which could alleviate oxidative-stress-associated kidney injury [42]. Indeed, AFB_1_-induced tissue injury was mediated through oxidative reaction [28]; (2) Se could reduce the formation of DNA adducts of aflatoxin in the chick [43,44]; and (3) Se could attenuate the decreased activity of Na^+^-K^+^ ATPase induced by AFB_1_. Our result showed that Se could elevate AFB_1_-depressed Na^+^-K^+^ ATPase activity which was accordance with the recent research [45]. Then, the reversal of the inbalance of Na^+^-K^+^ ATPase activity could ensure the cellular normal function. Therefore, 0.4mg/kg selenium supplemented as sodium selenite could appropriately protect the kidney from pathology lesions and functional changes induced by AFB_1_ exposure.

## 5. Conclusions

In conclusion, 0.3 mg/kg AFB_1_ could induce an increase in renal relative weight, cause pathological and ultrastructural changes, and have negative effects on renal functions. By contrast, 0.4 mg/kg supplemented selenium could ameliorate the AFB_1_-associated damages in kidney in chickens.
